# Pistol and Rifle Performance: Gender and Relative Age Effect Analysis

**DOI:** 10.3390/ijerph17041365

**Published:** 2020-02-20

**Authors:** Daniel Mon-López, Carlos M. Tejero-González, Alfonso de la Rubia Riaza, Jorge Lorenzo Calvo

**Affiliations:** 1 Departamento de Deportes de la Facultad de Ciencias de la Actividad Física y del Deporte-INEF de la Universidad Politécnica de Madrid, 28040 Madrid, Spain; 2 Department of Physical Education, Sport and Human Movement, Autonomous University of Madrid, 28049 Madrid, Spain

**Keywords:** Olympic shooting, elite, World Championship, shooter, RAE, precision, female

## Abstract

Background: The sport overrepresentation of early-born athletes within a selection year is called relative age effect (RAE). Moreover, gender performance differences depend on the sport. The main objectives of the study were to compare performances between gender and RAE in precision shooting events. Method: The results of 704 shooters who participated in the most recent World Shooting Championship were compared. Performance was analysed by event (rifle and pistol), gender and category (junior and senior), together with RAE and six ranges of ranking positions. Results: The results of the study indicated that men scored higher than women in pistol events and that no performance differences were found in rifle events when the whole group was compared. According to the birth trimester, no significant differences were found in the participant’s distribution, nor in performance in any case. Conclusions: The main conclusions of the study are: (1) the men’s pistol performance is better than the women’s even though RAE is not associated to the shooting score in any case; (2) men and women performed equally in the general analysis, but their performances were different depending on category and event with no RAE influence.

## 1. Introduction

Thousands of people practice shooting activities in the world as policemen, soldiers, shooters or hunters. Shooting is a precision activity, which needs maximum levels of motor control to hit the target. Minimum percentages of points/hits are the difference between success and failure for Olympic shooters. Consequently, the performance of precision events is measured by the championship sum of points, with the winner being the shooter who reaches the highest score [1]. 

Recently, the International Shooting Sport Federation (ISSF) has changed the shooting sport rules, matching the number of shots between men and women [1] following the IOC committee advice regarding gender equality in sports [2]. This change was analysed by Mon-López et al. [3] who concluded that the increase in shots did not mean a decrease in women’s sports performance. However, the need to investigate shooting performance in depth was discussed, not only to analyse global samples but to analyse the subsamples of a range of competition results as other authors have previously suggested [4]. 

Regarding the shooting performance comparison between men and women, Mon-López, Tejero-González and Calero [3] revealed that men obtain better results in pistol shooting than women; however, there were no performance differences between men and women in rifle shooting. Similarly, Goldschmied and Kowalczyk [4] found no differences between gender in Olympic rifle events (air rifle and small-bore rifle), and Kemnitz et al. [5] found no differences in military rifle shooting. Contrary to these results, men performed better than women in other sports [6], and specifically in shooting, women fatigue more than men when firing with certain types of rifles [7]. Moreover, policemen performed better than policewomen with guns [8], and men performed better with 9mm pistols than women [9]. However, these results contrast with those of Vučković et al. [10] who only found performance differences between genders at the beginning of a pistol training programme.

According to the ISSF rules and regulations, shooters are grouped in categories by chronological age (born after January 1) [1]. This rule could cause a disadvantage (unequal sports development) for those born in the last months of the year due to the maturational age being obviated in the talent identification and development (TID) systems. Therefore, this kind of sport policy seems not to respect the personal, individual and unique way that every athlete has to grow and evolve over the years [11]. This phenomenon, known as relative age effect (RAE) [12], could cause that the athletes considered relatively older to have more opportunities to evolve in their sports career and, therefore, get better individual performances [13] due to different factors: biological [14], social [15], geographical [16], etc.

The RAE has been demonstrated in numerous sports disciplines. A RAE meta-analysis in 14 sports showed that the number of athletes born in the first quartile (Q1) of the year was greater than those born in the last quartile (Q4) with a minimum proportion of three born in Q1 to two born in Q4 [17]. However, this effect has occurred more times in the field of team sports [18] than in individual sports [19].

Physical demands do not appear to be as decisive in shooting as in other individual sports [3,4]. For this reason, it could be thought that RAE is not a key factor for elite shooters and top championship participation as it is in other sports [20]. Furthermore, Delorme and Raspaud [21] showed that RAE was not found in women shooters, except in the 15–17 year group. However, impact of the RAE was stronger in the male adult, between 15–17, 11–12 and under 11 year groups. This weak association between RAE and participation, especially in women’s sport contexts, could be due to the development of sports practice in contexts that emphasise technical skills over physical factors [22]. Unfortunately, Delorme and Raspaud [21] only analysed the participation and not the performance differences and suggested that future studies clarify their results.

According to TID systems, the RAE is an important factor to select players in most sport disciplines where the physical attributes can be an advantage [23]. However, in individual sports, such as shooting, in which the weapon used in young official competitions can be adapted in size, weight and some aids are permitted [1], and the selection of athletes is less influenced by maturational and development reasons (RAE) as in team sports [18]. Therefore, there are no clear previous selection biases in the youth categories. However, Romann et al. [24] suggested that talent identification models in men in Switzerland are biased by RAE due to relatively older shooters having had slightly more opportunities to be selected within the Swiss Talent Development Programme (STDP) than their younger counterparts. Thus, real impact of the RAE on TID systems in shooting sports is still unclear.

Regarding women and RAE, overall pooled results have been published in small sport contexts. Thus, RAE was higher in ≤11 year and 12–14 year groups at higher competition levels [25]. In contrast, other authors suggested that there is no RAE in elite women sports [12,26] due to the great depth of competition hypotheses [18] and other sociocultural factors in female contexts [27].

To reduce RAE in individual sports, some strategies have been proposed such as a later sports specialization and limiting the achievement of performance at early ages [28]. Another approach could be grouping athletes by their level of maturational development or bio-banding, which would assure a decrease in performance differences and RAE [29]. However, there seems to be no agreement on which strategy would be the most appropriate to reduce RAE on an athlete’s participation and performance in official competitions.

According to the literature, there is limited knowledge on gender influence on performances in pistol and rifle events, especially when performance is analysed by competition range positions, where the need for more studies has been suggested previously [3]. Furthermore, as far as we know, there are no in-depth studies that have analysed the relationship between RAE and elite shooters’ performances. On the other hand, there are some doubts about whether RAE is a key factor in shooters’ selection for international competitions or if this aspect influences performance based on gender and competition category. Consequently, the main objectives of the study were: (1) to confirm whether men and women have performance shooting differences and (2) to explore the relationship between RAE and shooting performance.

## 2. Materials and Methods

### 2.1. Participants

The participants of the study were 704 shooters who participated in the World Shooting Championship held in Korea in 2018. Table 1 illustrates the participants’ distribution during the competition: 48.2% pistol and 51.8% rifle; 49.6% men and 51.4% women; 37.8% junior category, shooters under 21 years [1] and 62.2% senior category.

### 2.2. Method

Each athlete made six sets of 10 shots. The score of each shot was estimated by a Sius Ascor electronic target technology according to the ISSF rules [1,30]. Shooting competition performance was analysed using the sum value of all sets (60 shots), which implies a theoretical performance range between 0 and 654 points in the rifle event and 0 to 600 in the pistol event. In addition, all athletes were included in one of the following six ranking position ranges depending on the result they achieved in the tournament: positions 1st to 10th, 11st to 20th, 21st to 30th, 31st at 40th, 41st to 50th and 51st to last. The present study was approved by the Research Ethics Committee of the Polytechnic University of Madrid.

### 2.3. Data Analysis

The data were described with the arithmetic mean (*M*) and the standard deviation. They were analysed with *T*-test and ANCOVA (where the variable year of birth was controlled or adjusted in order to analyse the RAE). A Chi-squared test was used to analyse the sample distribution by birth trimester. The level of significance was set at *P* < 0.050. When statistically significant differences were found, on the one hand, the effect size was estimated using the delta parameter (*δ*), establishing three cut-off points: low effect (0.20), medium effect (0.50) and large effect (0.80); on the other hand, the performance difference in terms of percentage was calculated (*% =* $\frac{M1-M2}{M1}\times \;100$). IBM SPSS Statistics software (Version 25.0. IBM Cor) was used to make the mathematical estimation.

## 3. Results

The pistol shooting performance contrast between men and women is outlined in Table 2. It was observed that men scored higher than women in both junior category, where men scored 564.293 and women 560.471 points (*P* = 0.016, *δ* = 0.434, % = 0.677), as well as in the senior category, where men reached 570.400 and women 564.633 points (*P* < 0.001, *δ* = 0.601, % = 1.011). In the same way, when ranking positions were contrasted, differences were found in four of the six ranking ranges in the junior category and five ranking ranges in the senior category, always favourable for men, who achieved higher scores than women with large effect sizes between 1.245 and 11.405 and performance percentage differences between 0.189% and 1.637%.

Table 3 illustrates the performance contrasts between men and women in the rifle event. Considering all shooters, no statistically significant differences were found in the junior category (*P* = 0.760), where men scored 618.894 points and women 618.580, or in the senior category (*P* = 0.114), where the average of men was 619.687 points and women 621.128. When the contrasts were made by ranking position ranges and significant differences were found, women achieved higher scores than men in four of the six ranking ranges in the junior category, with large effect sizes between 1.494 and 5.028 and performance percentage differences between 0.262% and 0.447%. However, in the senior category, there were two ranks where women reached higher scores (the 41–50 and 51 to last position ranges) and two ranges where the men had better performance (the 11–20 and 21–30 position ranges).

Table 4 shows a balanced distribution of participants by birth trimester. There were no statistically significant differences in any case (all *P* > 0.112).

Table 5 reveals the performance contrasts according to the birth trimester. Comparisons were made by gender, category, event and the combination of these variables, controlling (or adjusting) the year of the birth of participants in order to analyse the RAE. No statistically significant differences were found in any case (all *P* > 0.202).

## 4. Discussion

The data report two main findings: (1) men’s pistol performance is better than women’s even though the relative age is not associated to shooting performance in any case, and (2) the data are inconclusive in the rifle event. On the one hand, no differences were found between men and women when all participants were compared. On the other hand, the junior category analyses carried out by ranking positions showed a better performance of women, while in the senior category, women or men performed better depending on the ranking position.

Performance differences in absolute values between genders have been demonstrated several times in many sports. Normally, these performance differences (around 10%) are due to the genetic profile, which implies hormonal, anthropometrical and physiological differences [6]. In Olympic shooting, after the last regulatory changes made by the ISSF following the IOC committee advice regarding gender equality in sports [1,2] the need to investigate shooting performance in depth has been suggested [3,4].

Regarding the shooting performance comparison between men and women, our results suggest that men performed better than women in the pistol event in both the junior and senior categories. Specifically, in the junior category, men outperformed women until position 40. In the senior category, men scored better than women except for the top 10 positions. Hence, these results are in accordance with Mon-López, Tejero-González and Calero [3] who show that men obtain better results in the pistol event than women. Moreover, in other disciplines such as police shooting, men outperformed women [8] as well as with other calibres like 9mm pistol, where men performed better than women [9]. Contrary to these results, Vučković, Dopsaj, Radovanović and Jovanović [10] only found performance differences between genders at the beginning of a pistol training programme. These performance differences could be due to the strength differences between genders in finger flexor [9] and shoulder abductor muscles [8] essential to stabilise the pistol.

However, in rifle shooting, no differences were found between men and women when the entire groups were compared, but differences were found favourable to women in the junior category between ranking positions 11 to 50 and in the senior category above position 41. On the other hand, men reached higher levels of performance in rifle shooting between positions 11–30. Consequently, these results were in line with the studies of Goldschmied and Kowalczyk [4] and Mon-López, Tejero-González and Calero [3] when general groups were compared. Furthermore, other studies in rifle military events showed no performance differences based on gender [5]. 

Despite the aforementioned data, it is interesting to see how the results were different when the analyses were done by ranking position ranges in rifle, especially in the junior category, where women outperformed men in four out of six position ranges, which is the opposite to what happens in other sports [6]. Moreover, these results could indicate that air rifles do not generate greater fatigue in women, unlike what happened in the study of Kemnitz, Rice, Irwin, Merullo and Johnson [7]. Therefore, there might not be a reduction in women’s body stability [31]. Another possible explanation for the absence of rifle performance differences could be that physical demands are smaller in shooting than in other sports [4]. Consequently, skills like movement accuracy and coordination could be key factors in shooting [32,33], specifically when balance seems to play a stronger role in rifle than in pistol events [34]. 

The results of our study showed no RAE in any case. This phenomenon could be partially explained due to the low importance of physical and anthropometric factors in shooting compared to other sports [3,4]. Thus, sport disciplines that need greater mastery of motor skills and techniques over physical capacities showed no RAE [35]. Moreover, another reason that RAE was not found in shooting could be the reduced number of sport dropouts, which could be different between shooting and other sports [36], giving shooters opportunities to reach higher performance levels regardless of their chronological age. Surprisingly, another reason to explain the absence of RAE in shooting could be the lack of talent detection programs in young athletes when shooting is compared with other sports. This lack could be partly explained due to the differences in the number of licenses, the lower level of economic resources and the scarcity of scientific articles in this field.

Regarding the impact of gender and birth date on RAE, our percentage distribution of participants was as expected in terms of events (pistol and rifle) and in terms of gender. These results were contrary to the findings of Delorme and Raspaud [21], who evidenced the presence of RAE in some male categories discontinuously. Nevertheless, both studies highlighted the lack of the RAE in female shooting competitions. Similar results were found in individual sports that are based on technical and motor control [37]. In contrast, opposite results were found in other studies on collective [18] and individual sports with marked physical and/or anthropometric character [19]. Again, maturation factors could have little relevance, and the impact of RAE could be less in TID systems of individual sports based on technical execution and precision to achieve high performance [35].

No performance shooting differences were found depending on the date of birth. Moreover, when absolute average values of performance between pistol and rifle events were compared, an inverse RAE trend was observed in relation to sport performance in male senior rifle shooters. The shooters born in Q4 earned better scores than those born in Q1 (without Q4 over-representation). The explanation for this finding could be in the use of different weapons. A pistol requires shoulder and grip strength [38,39], while a rifle requires balance skills [32,40]. This implies that the younger rifle shooters in the senior category compete under lower physical demands regardless of the maturational development associated with chronological age. It seems that technical and motor factors could be more important than conditional ones.

These results are in accordance with other sports, such as handball [41] where no relationship was demonstrated between the chronological age of the athletes and the team’s success. However, in other studies, the play position and team performance were affected by the RAE [42]. Although these are team studies, the relationship between the RAE and performance could be explained because the participants belonged to lower categories, and sometimes men and women were mixed.

Although there are a few studies that analyse the shooting performance differences between elite men and women shooters, there is not much literature that can be found regarding the RAE in Olympic shooting. Some limitations of this study should be mentioned. Our data set is limited to the last World Championship, which implies a small sample of top elite precision shooters. Therefore, future cross-sectional and longitudinal studies with larger samples of different performance levels and different age categories and that include non-precision events, such as trap shooting or skeet, would provide important additional information on the RAE and gender shooting performance differences. Lastly, future research analysing how physical factors affect performance by competition range positions could be interesting in precision modalities like air pistol.

## 5. Conclusions

It is important to mention that this study could have some practical applications. Coaches should make no differences in their training programs depending on the RAE in the junior category. Furthermore, different training strategies by gender should be implemented in pistol but not in rifle events, where the level of the shooter’s performance should guide the type of training. Lastly, it could be said that men have better scores than women in pistol shooting, but not in rifle shooting, where performance depends on category and the range of ranking positions. Additionally, the RAE seems not to have any effect on shooting performance.

## Figures and Tables

**Table 1:** Participants description.

Sample		Age (Years)	Pistol and Rifle		Pistol		Rifle	
			*N*	*%*	*N*	*%*	*N*	*%*
All		24.810 ± 8.063	704	100	339	48.200	365	51.800
Gender	Men	25.980 ± 8.499	349	49.600	173	51.000	176	48.200
	Women	23.650 ± 7.500	355	50.400	166	49.000	189	51.800
Category	Junior	18.350 ± 1.383	266	37.800	126	37.200	140	38.400
	Senior	28.730 ± 7.917	438	62.200	213	62.800	225	61.600

Annotations: Age column = arithmetic mean of the years of life ± standard deviation; N = number or frequency; % = Percentage.

**Table 2:** Performance contrast between men and women in pistol event.

Category	Position	Pistol				
		Men	Women	*P*	*δ*	%
Junior and Senior	All	568.353 ± 9.094	562.928 ± 9.791	<0.001	0.574	0.955
Junior	All	564.293 ± 9.767	560.471 ± 7.706	0.016	0.434	0.677
	1–10	577.400 ± 3.747	570.700 ± 2.331	<0.001	2.147	1.160
	11–20	570.000 ± 0.816	566.900 ± 0.994	<0.001	3.408	0.544
	21–30	567.200 ± 0.918	563.600 ± 0.843	<0.001	4.084	0,635
	31–40	563.700 ± 1.767	560.900 ± 1.197	0.001	1.855	0.497
	41–50	557.000 ± 1.563	557.400 ± 1.264	0.537	-	-
	≥51	547.000 ± 5.237	550.944 ± 5.954	0.119	-	-
Senior	All	570.400 ± 8.030	564.633 ± 10.915	<0.001	0.601	1.011
	1–10	582.400 ± 1.955	581.300 ± 3.128	0.358	-	-
	11–20	579.200 ± 0.788	573.800 ± 0.918	<0.001	6.312	0.189
	21–30	576.600 ± 0.516	572.100 ± 0.875	<0.001	6.264	0.780
	31–40	574.500 ± 0.707	570.200 ± 0.788	<0.001	5.744	0.748
	41–50	573.300 ± 0.483	567.600 ± 0.516	<0.001	11.405	0.994
	≥51	565.169 ± 6.462	555.917 ± 8.284	<0.001	1.245	1.637

Symbology: *P* = *T*-test statistical significance probability for independent samples, *δ* = delta effect size, *%* = percentage performance difference. Note: In the cells of the men and women columns, the arithmetic mean of sports performance is indicated (maximum theoretical value of 600 points) ± standard deviation.

**Table 3:** Performance contrast between men and women in rifle event.

Category	Position	Rifle				
		Men	Women	*P*	*δ*	%
Junior and Senior	All	619.398 ± 7.344	620.104 ± 5.875	0.311	-	-
Junior	All	618.894 ± 5.371	618.580 ± 6.761	0.760	-	-
	1–10	626.920 ± 1.348	627.010 ± 1.653	0.893	-	-
	11–20	622.560 ± 1.374	624.190 ± 0.701	0.003	1.494	0.262
	21–30	619.580 ± 0.492	622.350 ± 0.604	<0.001	5.028	0.447
	31–40	618.550 ± 0.365	620.540 ± 0.739	<0.001	3.414	0.332
	41–50	616.360 ± 0.856	618.010 ± 0.944	0.001	1.831	0.268
	≥51	611.914 ± 4.315	611.196 ± 5.502	0.675	-	-
Senior	All	619.687 ± 8.272	621.128 ± 4.969	0.114	-	-
	1–10	629.420 ± 1.891	628.290 ± 1.582	0.165	-	-
	11–20	626.500 ± 0.573	625.960 ± 0.464	0.004	1.035	0.086
	21–30	625.480 ± 0.253	624.900 ± 0.230	<0.001	2.398	0.093
	31–40	624.280 ± 0.410	624.130 ± 0.258	0.341	-	-
	41–50	622.690 ± 0.369	623.230 ± 0.483	0.012	1.256	0.087
	≥51	614.834 ± 8.128	617.816 ± 4.064	0.011	0.464	0.485

Symbology: *P* = *T*-test statistical significance probability for independent samples, *δ* = delta effect size, *%* = percentage performance difference. Note: In the cells of the men and women columns the arithmetic mean of sports performance is indicated (maximum theoretical value of 654 points) ± standard deviation.

**Table 4:** Frequency or percentage of participants by birth trimester.

Sample	Birth Trimester								*P*
	Q1		Q2		Q3		Q4		
	*N*	*%*	*N*	*%*	*N*	*%*	*N*	*%*	
All	182	25.900	169	24.000	183	26.000	170	24.100	0.910
Pistol									
Junior	31	24.600	37	29.400	30	23.800	28	22.200	0.699
Senior	46	21.600	48	22.500	63	29.600	56	26.300	0.330
Men	38	22.000	45	26.000	48	27.700	42	24.300	0.737
Women	39	23.500	40	24.100	45	27.100	42	25.300	0.918
Rifle									
Junior	42	30.000	24	17.100	41	29.300	33	23.600	0.112
Senior	63	28.000	60	26.700	49	21.800	53	23.600	0.535
Men	51	29.000	41	23.300	40	22.700	44	25.000	0.641
Women	54	28.600	43	22.800	50	26.500	42	22.200	0.554

Symbology: Q1 = Quartile 1, players born between 1 January and 31 March; Q2 = Quartile 2, players born between 1 April and 30 June; Q3 = Quartile 3, players born between 1 July and 30 September; Q4 = Quartile 4, players born between 1 October and 31 December; *P* = Statistical significance probability in Chi-squared test.

**Table 5:** Performance contrasts by trimester of birth.

Sample	Birth Trimester				*P*
	Q1	Q2	Q3	Q4	
Gender					
Men	597.049 ± 26.285	592.445 ± 27.639	592.293 ± 26.172	594.530 ± 27.514	0.734
Women	597.190 ± 28.490	593.025 ± 29.263	591.745 ± 30.669	591.310 ± 30.328	0.679
Category					
Junior	594.901 ± 27.401	585.036 ± 28.363	594.289 ± 30.791	592.669 ± 30.028	0.202
Senior	598.608 ± 27.355	597.076 ± 27.555	590.563 ± 27.022	593.090 ± 28.386	0.390
Event					
Pistol	566.403 ± 8.570	565.788 ± 9.294	565.462 ± 10.639	565.214 ± 10.773	0.685
Rifle	619.649 ± 6.166	619.993 ± 6.746	619.440 ± 6.991	620.019 ± 6.749	0.914
Pistol					
Junior	564.258 ± 7.289	563.081 ± 8.535	559.800 ± 10.141	561.464 ± 9.283	0.206
Senior	567.848 ± 9.128	567.875 ± 9.400	568.159 ± 9.847	567.089 ± 11.050	0.955
Pistol					
Men	567.947 ± 8.837	567.467 ± 9.104	569.917 ± 9.305	567.881 ± 9.176	0.605
Women	564.897 ± 8.133	563.900 ± 9.253	560.711 ± 9.974	562.548 ± 11.670	0.244
Rifle					
Junior	617.519 ± 6.758	618.883 ± 3.698	619.524 ± 6.304	619.145 ± 6.565	0.403
Senior	621.068 ± 5.333	620.437 ± 7.615	619.369 ± 7.583	620.562 ± 6.866	0.789
Rifle					
Men	618.733 ± 5.915	619.861 ± 7.733	619.145 ± 8.245	619.968 ± 7.774	0.816
Women	620.513 ± 6.326	620.119 ± 5.741	619.676 ± 5.878	620.071 ± 5.572	0.912

Symbology: Q1 = Quartile 1, players born between 1 January and 31 March; Q2 = Quartile 2, players born between 1 April and 30 June; Q3 = Quartile 3, players born between 1 July and 30 September; Q4 = Quartile 4, players born between 1 October and 31 December; *P* = Statistical significance probability in ANCOVA with adjustment for the variable year of birth. Note: The birth trimester columns indicate the arithmetic average of performance (maximum theoretical value of 654 points/rifle and 600 points/pistol) ± standard deviation.
